# Zoledronic acid prevents the tumor-promoting effects of mesenchymal stem cells via MCP-1 dependent recruitment of macrophages

**DOI:** 10.18632/oncotarget.4658

**Published:** 2015-08-03

**Authors:** Xiao-Hua Jia, Yang Du, Duo Mao, Zhong-Liang Wang, Zhen-Qiang He, Jing-Dan Qiu, Xi-Bo Ma, Wen-Ting Shang, Dan Ding, Jie Tian

**Affiliations:** ^1^ Key Laboratory of Molecular Imaging of the Chinese Academy of Sciences, Institute of Automation, Chinese Academy of Sciences, Beijing 100190, China; ^2^ State Key Laboratory of Medicinal Chemical Biology, Key Laboratory of Bioactive Materials, Ministry of Education, College of Life Sciences, Nankai University, Tianjin 300071, China; ^3^ School of Life Science and Technology, Xidian University, Shaanxi, Xi'an 710071, China; ^4^ State Key Laboratory of Oncology in South China, Department of Neurosurgery, Sun Yat-sen University Cancer Center, Guangzhou 510060, China; ^5^ Department of General Surgery, the Chinese PLA General Hospital, Beijing 100039, China; ^6^ Beijing Key Laboratory of Molecular Imaging, Beijing 100190, China

**Keywords:** zoledronic acid, mesenchymal stem cells, breast carcinoma, tumor associated macrophages, monocyte chemotactic protein-1

## Abstract

Zoledronic acid (ZA) has been tested in clinical trials as an additive therapy for early-stage breast cancer. However, the mechanism by which ZA exerts its antitumor activity is still unclear. The aim of this study is to investigate whether the prevention of tumor growth by ZA is through regulating the mesenchymal stem cells (MSC)-monocyte chemotactic protein 1 (MCP-1)-macrophages axis in the tumor microenvironment.

To address this issue, MDA-MB-231-FLUC human breast cancer cells were cultured and injected either alone, or coupled with MSC into the mammary fat pads of nude mice. MSC were treated with either ZA or untreated. Tumor growth was determined by using an *in vivo* bioluminescence imaging (BLI) and the tumor-associated macrophages (TAMs) in tumor tissues were immunohistochemically analyzed by using CD206 antibody. The effects of ZA on the cytokine related gene expression of MSC were assessed by using real-time PCR.

In this study, we found that ZA-treated mice showed a significant delay in tumor growth. In addition, our data revealed that ZA weakened the ability of MSC to promote tumor growth by impairing TAMs recruitment and tumor vascularization. Furthermore, it was found that ZA decreased MCP-1 expression of MSC, and therefore reduced the recruitment of TAMs to the tumor sites and hence inhibited the tumor growth.

Altogether, our study demonstrated ZA can prevent the tumor-promoting effects of MSC. The antitumor effects of ZA were caused by decreasing the MCP-1 expression of MSC, which further decreased the infiltration of TAMs into tumor sites, and therefore inhibited the tumor growth.

## INTRODUCTION

Breast cancer continues to be the most common lethal malignancy diagnosed in female. Breast cancer tissues are composed of carcinoma cells and noncancerous stromal cells, including endothelial cells, immune cells, MSC and macrophages [1]. Crosstalk between epithelial breast cancer cells and stromal cells is important for progression of breast cancer [1]. Therefore, the anti-cancer therapy by targeting the stromal is a promising therapeutic option for the treatment of breast cancer.

MSC are a kind of non-cancerous stromal cells, which have the potential ability for self-renewal, long-term viability, and capacity for differentiation toward a variety of cell types [2]. Due to chronic inflammation in the tumor microenvironment, MSC are known to migrate to tumors, and differentiate into carcinoma-associated fibroblasts [3]. According to accumulating evidence, MSC could also have an adverse effect that favors tumor growth [4–7]. Moreover, the signaling pathway of interleukin-17B (IL-17B)/IL-17B receptor may mediate the interaction between human MSC and breast cancer cells [8]. MSC may sustain cancer cell growth and survival within the microenvironment, where they can contribute to the formation of “niches” for tumor growth [1]. Furthermore, conditioned medium of human MSC have been reported to promote proliferation, migration, and invasion of human breast cancer cells [4]. This suggests that tumorigenesis of breast cancer is acquired by paracrine signals from MSC within the tumor microenvironment. However, the paracrine signaling mechanisms by which MSC stimulates tumorigenesis are largely unknown.

The macrophages in tumor microenvironment were called tumor-associated macrophages (TAMs). TAMs could be induced emigration from bone marrow to the periphery by monocyte chemotactic protein 1 (MCP-1). MCP-1 is also indispensable in experimental autoimmune encephalomyelitis by MSC in mice, through suppressing the function of B cells and T cells [9]. Moreover, it has been found that through stimulating toll-like receptors in bacterial infections, bone marrow-resident MSC could produce abundant MCP-1 [10]. TAMs are known to possess the tumor promoting effects, which can be recruited by MCP-1. Previous studies showed that tumor resident MSC promoted tumor growth by recruiting monocytes/macrophages through MCP-1 [11]. Thus, the MSC-MCP-1-macrophages axis may be physiologically important in tumor progression.

ZA is a third-generation bisphosphonate. It has been used for the treatment of solid tumors, including prostate cancer, lung cancer, and colorectal cancer [12]. ZA has recently been tested as an additive therapeutic for early-stage breast cancer [13]. ZA also benefited patients with newly diagnosed multiple myeloma [14]. These findings clearly demonstrate the beneficial therapeutic effects of ZA on cancer patients. However, the mechanism underlying the antitumor activity of ZA remains unclear. Preclinical data obtained from *in vitro* and *in vivo* studies provide compelling evidences that ZA can inhibit multiple intracellular processes essential for cancer cell proliferation and invasion, and induce apoptosis [15]. Furthermore, clinically relevant doses of ZA exert profound effects on the host's anticancer response, such as inhibition of tumor-associated angiogenesis and modulation of macrophage phenotype, which may be important anticancer mechanisms *in vivo* [16]. However, the effects of ZA to noncancerous stromal cells such as MSC in tumor microenvironment remained unknown.

To assess the possible mechanism of ZA on antitumor activity, we analyzed the effects of ZA on the interaction between MSC and breast cancer cells. Firstly, we examined the effect of ZA on tumor growth in breast tumor bearing mice by using BLI, and found that the development of tumors was inhibited by ZA treatment. Then, we tested the effects of ZA treated MSC on tumor growth, and our results suggested that ZA treated MSC prevented the development of breast cancer *in situ*. Thirdly, we demonstrated that ZA impaired the tumor promoting capability of MSC by impairing TAMs recruitment. Furthermore, it was found that the antitumor effects of ZA-MSC were through decreasing the expression of MCP-1 of MSC, which further decreased the infiltration of TAMs. Altogether, this study demonstrated that ZA has antitumor capability and this biological activity was resulted from regulating the MSC-MCP-1-macrophages axis in the tumor microenvironment.

## RESULTS

### ZA treatment inhibited breast tumor growth

**Figure 1:**
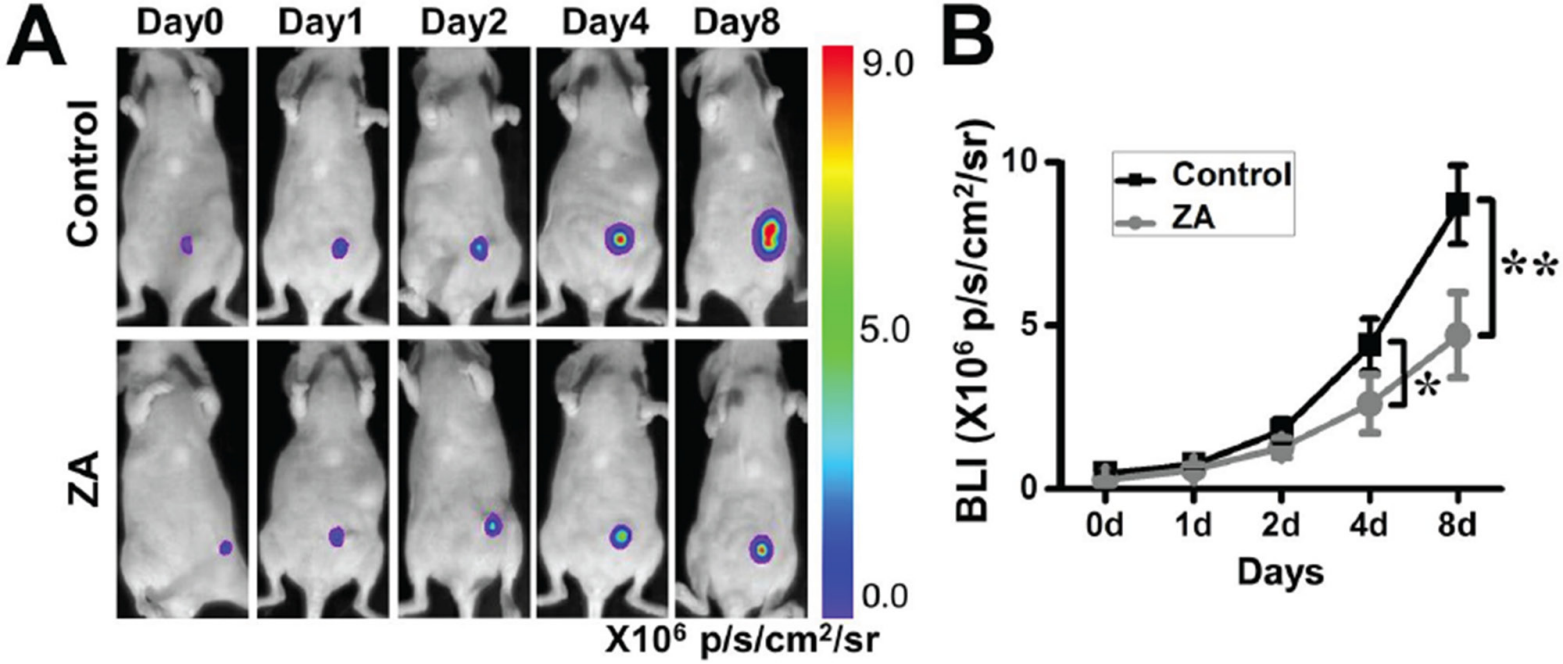
The antitumor function of ZA **A.** ZA-treated mice displayed a significant delay in the tumor growth rate. **B.** Quantification of the bioluminescence signal in tumors after different treatments. (*P < 0.05; **P < 0.01)

We firstly assessed the effect of ZA on tumor growth. ZA was administered intravenously to MDA-MB-231-FLUC breast tumor bearing mice, and the tumor growth was monitored by using BLI. The tumor growth rate in the ZA-treated group was less than the control group. On the fourth day, there were significant differences between the two groups. The results showed that the ZA treatment significantly decreased the BLI signal of tumors compared to the control mice (Figure 1A and 1B).

### Characterization of MSC

**Figure 2:**
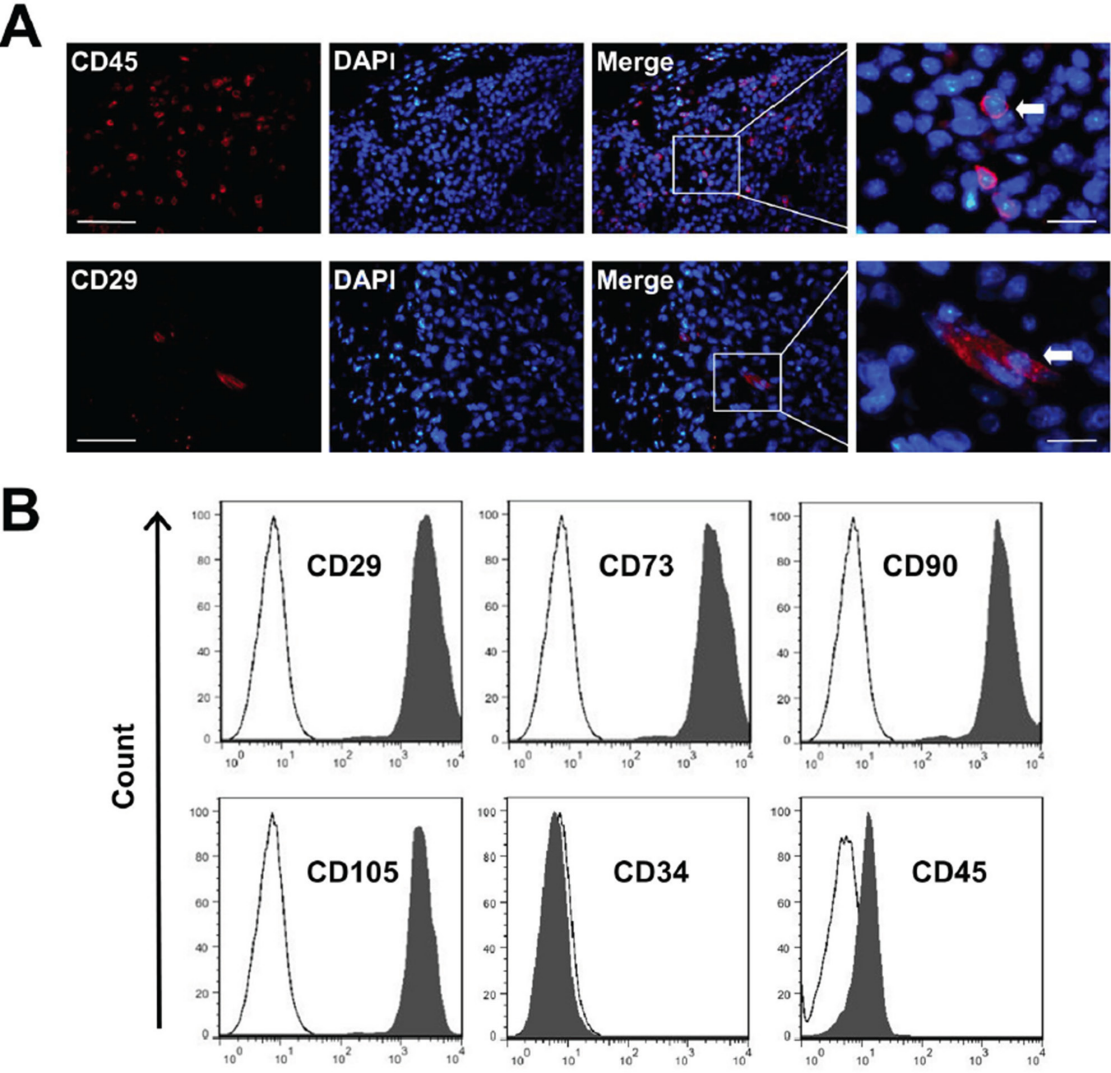
The characterization of MSC **A.** Frozen sections of breast carcinoma tumor were immunostained with anti-CD29 and CD45. CD29^+^ and CD45^+^ stem cells (arrow) migrated into the tumor site. Nuclei were stained with DAPI (blue). Scale bars = 50 μm (left) and 5 μm (right) respectively. **B.** Quantitative analysis of cell marker expression by FACS. MSC express high levels of CD29, CD73, CD90 and CD105, but almost negative for CD34 and CD45.

In this study, CD29-positive MSC and CD45-positive hematopoietic stem cells (HSCs) at the tumor sites were identified by using immunofluorescent staining (Figure 2A). Moreover, we cultured the MSC generated from mice bone marrow, and characterized them. Mice MSC showed a homogenous spindle-shaped morphology. MSC also were capable of differentiating into chondrogenic, adipogenic, and osteogenic cell lines, as evidenced by mast cells via toluidine blue staining, and mineralization of the MSC via Alizarin red S staining, respectively (data not shown). FACS was used to identify MSC surface marker expression. High expression level of CD29, CD73, CD90, CD105 and the low level of CD34 and CD45 were observed (Figure 2B), which was consistent with the previous report [17]. All results were replicated using at least three different MSC clones.

### ZA-treated MSC possessed less tumor-promoting capacity than untreated-MSC

**Figure 3:**
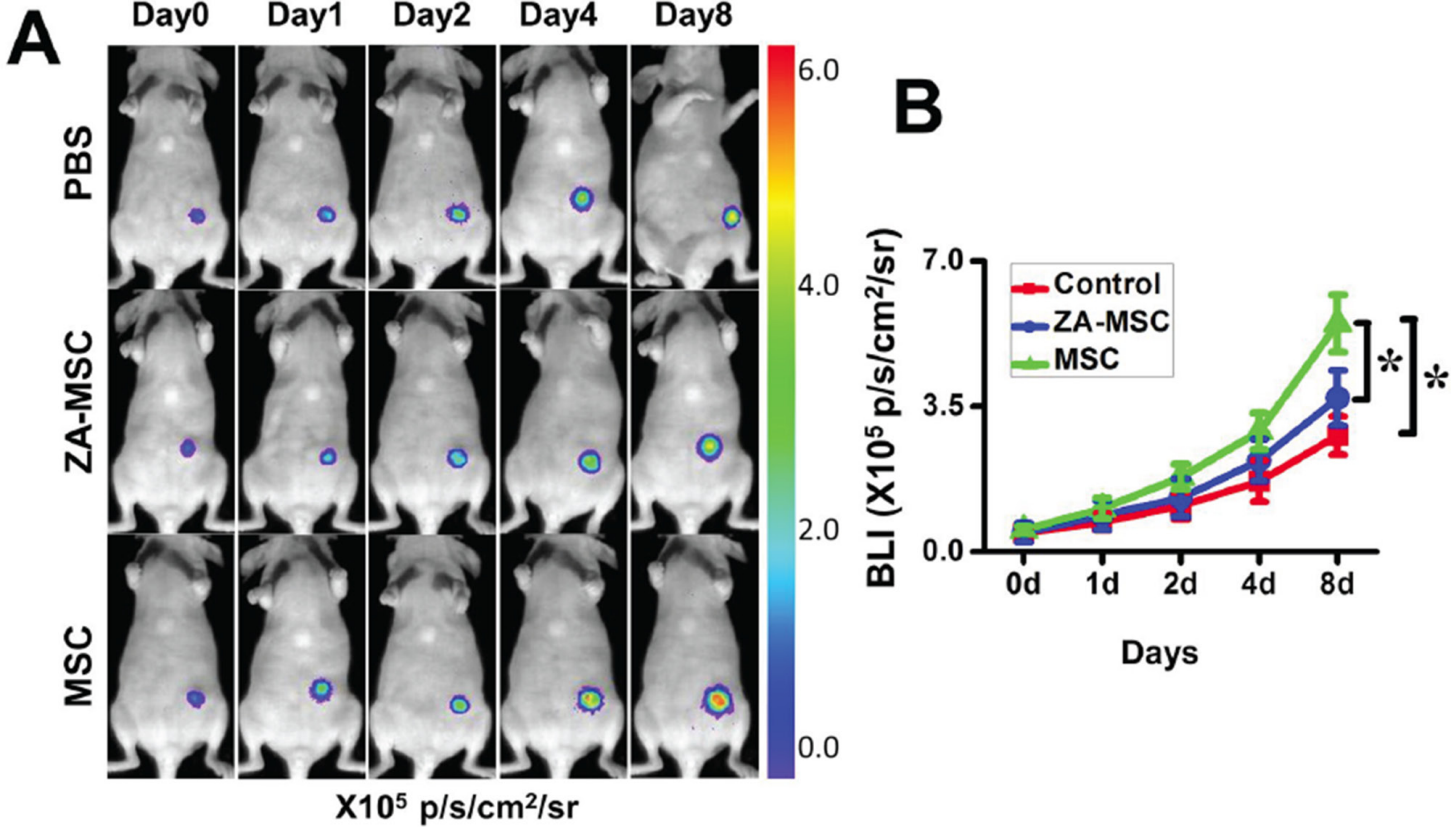
Less potential to promote tumor growth in ZA-MSC compared to untreated-MSC **A.** ZA-treated MSC showed a decreased ability to promote the breast tumor growth compared to untreated MSC. **B.** Quantification of the bioluminescence signal in tumors after different treatments. (*P < 0.05)

To determine the effects of ZA-treated MSC on tumor growth, we co-injected ZA-treated MSC and MDA-MB-231-FLUC human breast carcinoma cells into the mammary fat pads of nude mice. Untreated-MSC from the same wild-type mice were used as controls. Co-transplantation of MSC together with MDA-MB-231-FLUC cells significantly increased BLI signal of tumor xenograft compared to control group (PBS group) at day 8 (Figure 3A and 3B). However, co-injection of MDA-MB-231-FLUC cells and MSC in the presence of ZA resulted in slightly but not markedly increased BLI signal of tumors xenograft compared with the control group. Especially it was noted that co-transplantation of MDA-MB-231-FLUC cells, MSC and ZA significantly decreased tumor growth compared with the co-injection of MSC only at day 8 (Figure 3A and 3B).

### ZA-MSC treatment decreased TAMs migration and vascularization in the tumor sites

**Figure 4:**
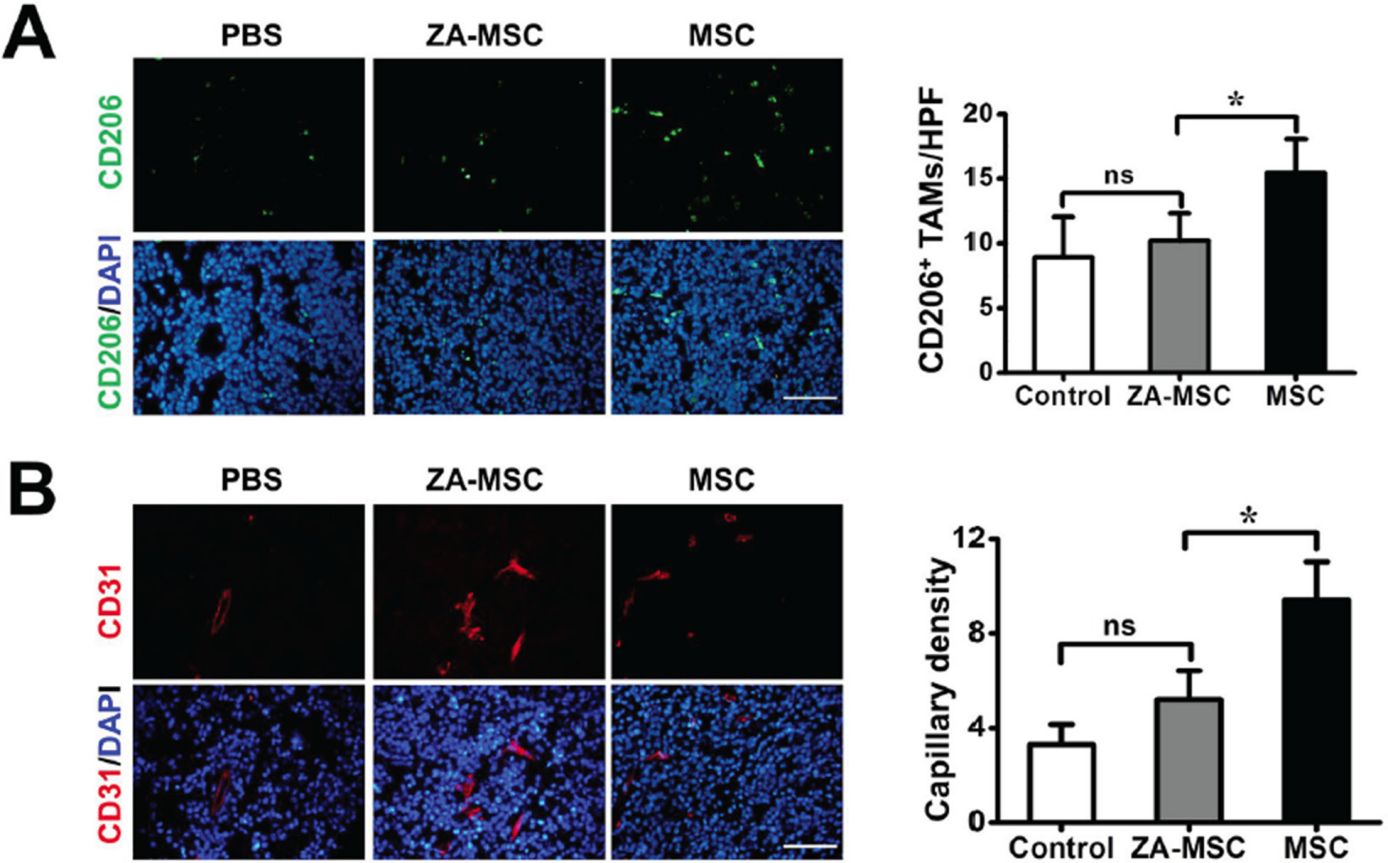
The analysis of neovascularization and TAMs migration of ZA-MSC treated tumor **A.** Immunohistochemical staining for CD206 (red) and DAPI (blue) in tumors 8 d after treatment. Scale bars = 50 μm. Less CD206 positive macrophages were stained in the ZA-MSC implantation group than MSC alone group. (*P < 0.05) **B.** Immunohistochemical staining for CD31 (red) and DAPI (blue) in tumors 8 d after treatments. Scale bars = 50 μm. Quantitative analysis revealed ZA-MSC administration could significantly decrease capillary density compared to MSC group. (*P < 0.05) Abbreviations: HPF, high-power field.

To determine whether ZA-MSC treatment affected TAMs infiltration, we used CD206 immunostaining to evaluate TAMs at the tumor site. We found that a less number CD206 positive macrophages were stained in the ZA-MSC implantation group and the PBS injection group than MSC alone. These results suggested that compared to untreated MSC, ZA-treated MSC significantly reduced CD206^+^ TAMs expression (Figure 4A). We also investigated the effect of ZA-MSC on tumor neovascularization by using CD31 marker, and found that ZA-MSC suppressed vascularization compared to the MSC (Figure 4B).

### ZA decreased the MCP-1 expression of MSC, and MCP-1 was involved in the tumor-promoting effect of MSC

**Figure 5:**
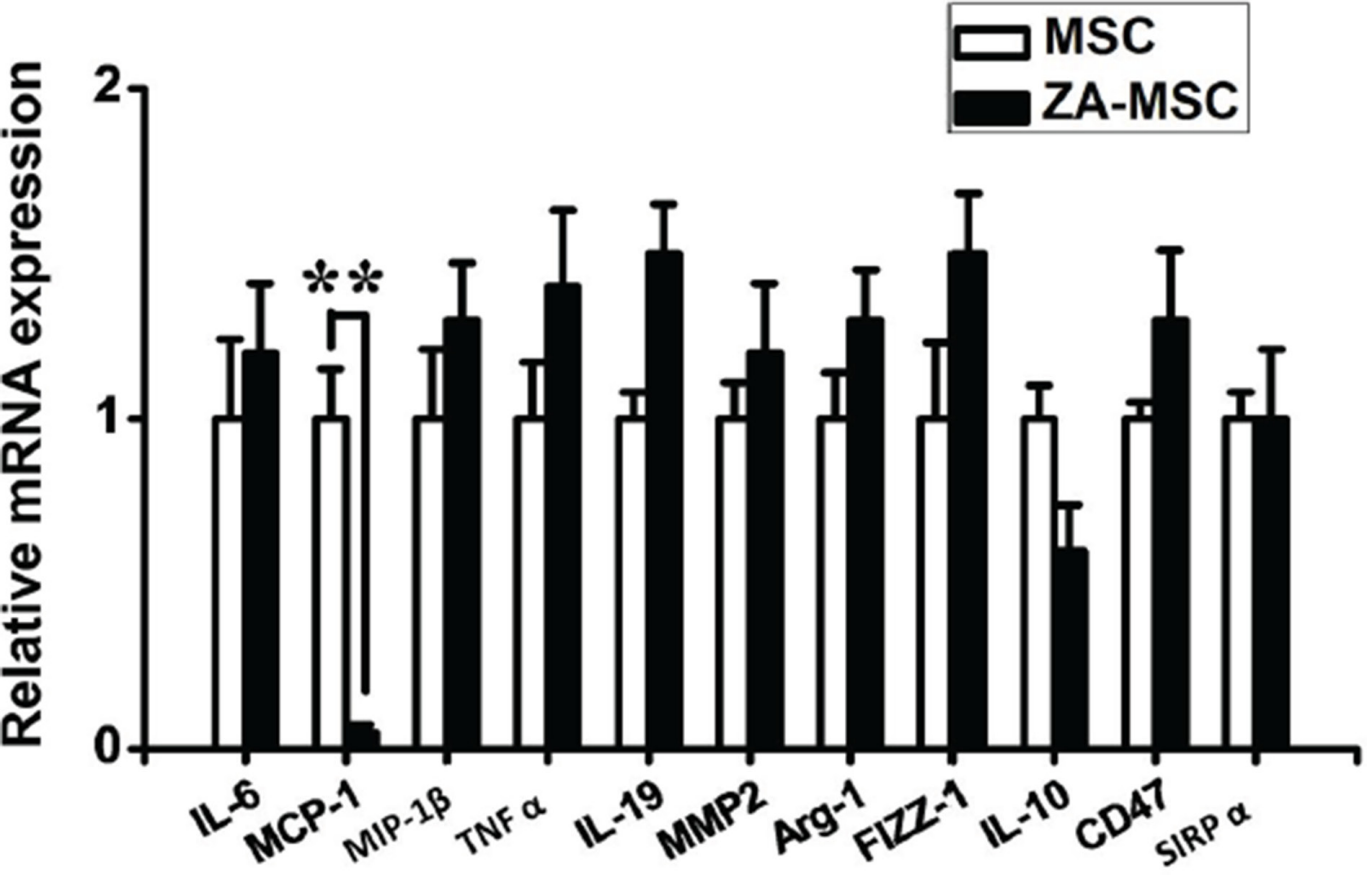
Effects of ZA on the gene expression in MSC A marginal reduction of the level of MCP-1 mRNA was detected in MSC after treatment with ZA. (*P < 0.05)

**Figure 6:**
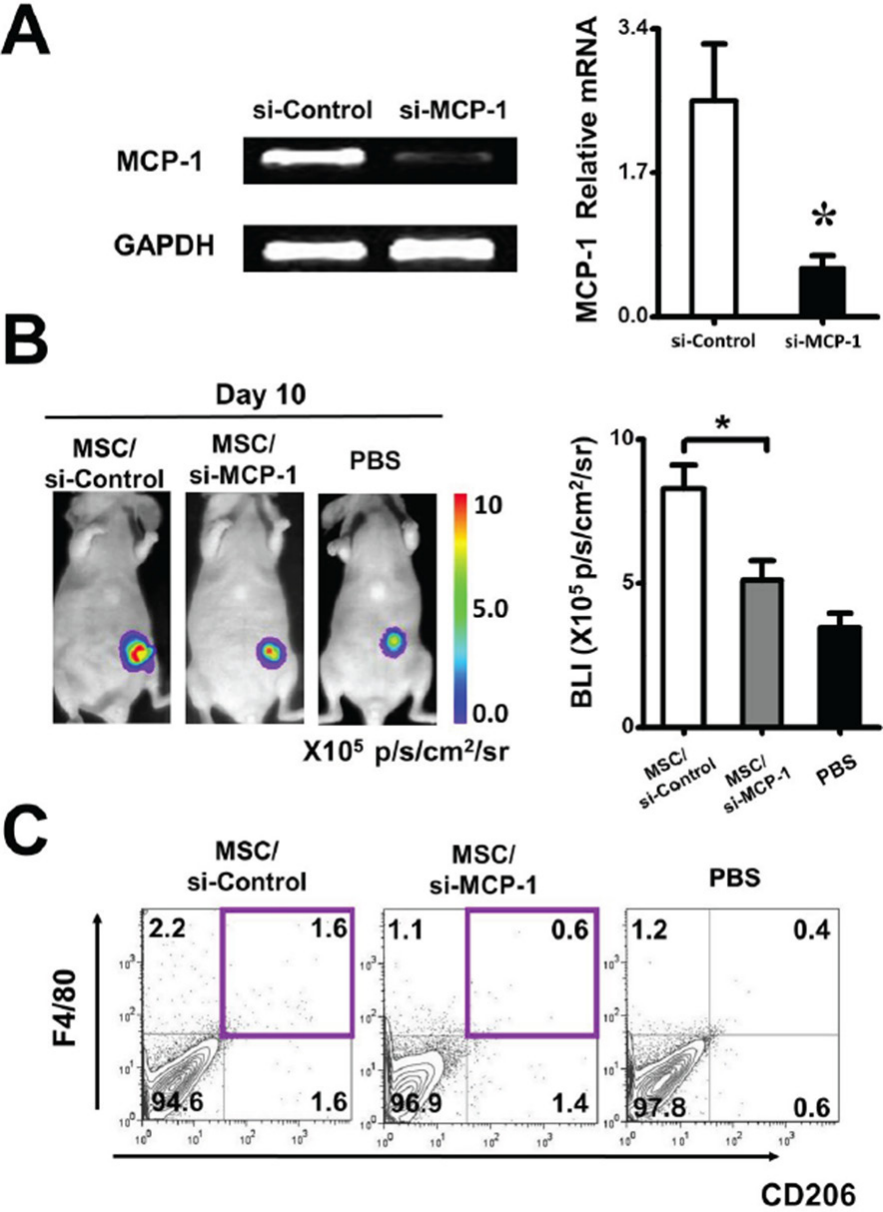
Regulation of tumor growth by ZA-MSC requires MCP-1 **A.** RT-PCR showed that the expression of MCP-1 was decreased in MSC in which transfections of MCP-1 siRNA were performed with a Lipofectamine. Meanwhile, real-time PCR result showed that the expression of MCP-1 was markedly decreased in MCP-1 siRNA treated MSC. The inhibitory efficiency was more than 86% compared with the control siRNA treated MSC. **B.** After MSC were treated by MCP-1 siRNA, the tumor promoting effect of MSC was weakened by MCP-1siRNA. **C.** Impact of MCP-1 on macrophages infiltration on tumor-bearing mice. Representative flow cytometry data show the frequency of macrophages from tumor with different treatment.

To further determine the effect of ZA on MSC, MSC were treated with 20 ng/ml ZA for 24 h. ZA significantly inhibited the MCP-1 gene expression in MSC, relative to the untreated MSC (Figure 5). To determine whether MCP-1was involved in affecting tumor growth of MSC, the expression of MCP-1 in MSC was downregulated by using MCP-1 siRNA. Real-time PCR was performed to confirm the downregulation efficacy of MCP-1. Compared with the control siRNA treated MSC, the expression of MCP-1 was markedly decreased in MSC treated with MCP-1 siRNA (Figure 6A). To investigate whether MCP-1 was indeed involved in the tumor-promoting effect of MSC *in vivo*, we firstly treated MSC with control siRNA or MCP-1 siRNA, then cotransplanted two kinds of MSC with MDA-MB-231-FLUC tumor cells into mice. We found that tumor growth was significantly reduced by MCP-1 siRNA treated-MSC compared to control siRNA-treated MSC (Figure 6B). Moreover, we evaluated macrophage infiltration percentage in recipient mice after coinjection of MSC and cancer cells by performing flow cytometry. FACS data showed that MCP-1 deficiency led to a significant reduction in CD206 positive macrophages numbers in tumor tissues on day 10 (Figure 6C).

## DISCUSSION

ZA is often administered as a combination treatment of cancer; however, the precise mechanism and contribution of ZA to the antitumor effect remain unknown. In this study, ZA-treated mice showed significantly delayed tumor growth. Moreover, our studies revealed that ZA diminished the ability of MSC to promote tumor growth by inhibiting TAMs recruitment and tumor vascularization. Furthermore, ZA decreased MCP-1 expression of MSC, and MCP-1 was involved in the tumor-suppressing effect of ZA-treated MSC. Altogether, our study suggested that the antitumor effects of ZA were through decreasing the expression of MCP-1 of MSC, and hence decreased the infiltration of TAMs, which are known to be involved in promoting tumor growth.

ZA is a bisphosphonate routinely used in the treatment of cancer-associated bone disease [18]. In cancer cells, ZA has been shown to cause apoptosis in both a caspase-dependent and caspase-independent manner, inhibiting the cell cycle, and reducing remodeling of the extracellular matrix and invasiveness [16]. In our study, we confirmed that ZA inhibited breast tumor progression *in vivo*. Cancer tissues are composed of carcinoma cells, endothelial cells, immune cells, MSC and macrophages [1]. Previous study showed that ZA treatment could inhibit the growth, migration, and vessel formation of endothelial cells [19]. ZA-treated bone marrow-derived macrophages also showed dose-dependent inhibition of proliferation and migration, and inhibited adhesion. Moreover, ZA inhibited the gene expression and the secretion of numerous growth factors from macrophages [20, 21]. However, there are rare studies focusing on investigating the effects of ZA on MSC in tumor sites.

Normal MSC adhere to matrix components, and as a result, home to the bone, lungs when injected intravenously [22]. Nevertheless, a growing number of studies have shown that MSC home to sites of injury induced by inflammation without organ specificity [23]. Previous studies also have shown that CD45^+^GFP^+^ bone marrow cells could infiltrate to various tissues [24]. MSC migration to tumors is due to the tumor microenvironment, consisting of soluble factors produced by inflammatory and tumor cells, and chemokine receptors on the MSC [17]. Carcinoma-associated fibroblasts originate from bone marrow and derive from MSC [25]. In our study, we found that CD29-positive MSC could present at the tumor sites. Recent studies revealed that bone marrow MSC and progenitor cells respond to circulating microbial molecules and induce monocyte emigration by secreting MCP-1 in proximity to bone marrow vascular sinuses [10]. MSC isolated from spontaneous lymphomas in mouse had tumor-promoting ability [11]. Our studies also revealed that MSC promoted tumor growth *in vivo*. However, ZA-treated MSC did not promote tumor growth *in vivo* as untreated-MSC. The results suggested that ZA-stimulated MSC possessed less tumorigenic potential of breast tumor cells *in vivo*. MSC have been shown to promote tumor growth and metastasis by regulating angiogenesis, tumor cell survival, immunosuppressive microenvironment shape, and cancer stem cell (CSC) maintenance, and mesenchymal niche construction [4]. In this study, we found that ZA-MSC suppressed the infiltration of TAMs and vascularization at the tumor site. These results strongly suggested that ZA impaired the ability of MSC to promote tumor growth by impairing TAMs recruitment and tumor vascularization.

Immunosuppression activities induced by TAMs are recognized as key mediators of tumor progression [26]. MCP-1, a potent monocyte attractant, binds to CCR2 receptors which mediated recruitment of monocytes/macrophages. Previous studies showed that MCP-1 was essential in suppression of B cell function and T cell function in experimental autoimmune encephalomyelitis by MSC in mice [9]. Our findings demonstrated that ZA inhibited the expression of MCP-1 by MSC *in vitro*. However, it remains unknown whether or not these effects are related to macrophage infiltration. To examine whether this antitumor effects of ZA-MSC were through decreasing the expression of MCP-1 of MSC and hence decreased the infiltration of TAMs, we further examined the TAMs expression by using CD206 immunostaining at the tumor sites. Our results showed that ZA treated MSC exhibited low MCP-1 expression, which indicated that MCP-1 was indeed involved in the antitumor effects of ZA-MSC.

The tumor microenvironment is complex, and it contains various cell types, soluble factors, extensive neovasculature, and excessive extracellular matrix deposition. The network orchestrated by tumor cells, stromal cells, and soluble factors contributes to tumorigenesis, progression, metastases, and reoccurrence [1]. Therefore further investigation is needed to fully understand the function and mechanism of ZA. MSC-MCP-1-macrophages axis is likely to be important in various pathological conditions. A better understanding of the underlying mechanisms can lead to better therapeutic application of ZA in clinics.

## MATERIALS AND METHODS

### Reagents

ZA was got from Sigma. Monoclonal rat anti-mouse antibodies against CD29, CD45, and CD31 were from BD Biosciences; and rat anti-mouse CD206 monoclonal antibodies were from Abcam. Alexa Fluor 594 and Alexa Fluor 488-conjugated donkey anti-rat secondary antibodies were from Invitrogen.

### Cell culture

The human breast carcinoma MDA-MB-231-FLUC cells were procured from the Department of Radiology, Peking Union Medical College Hospital, and were cultured in Dulbecco's modified Eagle' medium (Gibco) containing 10% fetal bovine serum (FBS), 10 U/ml penicillin, and 100 μg/ml streptomycin in a 5% CO_2_ atmosphere. Cells were passaged approximately every 3 d by trypsinization. MDA-MB-231-FLUC cells stably transfected with a lentiviral vector containing a firefly luciferase reporter gene were selected first *in vitro*, and then injected into immunodeficient nude mice.

MSC were collected from the bone marrow of the tibia and femur of mice aged 6–10 weeks. Cells were cultured in α-MEM medium (Invitrogen, Carlsbad, CA) supplemented with 10% FBS, 2 mM glutamine, 100 U/ml penicillin, and 100 μg/ml streptomycin (Invitrogen, Carlsbad, CA). Non-adherent cells were removed after 24 h, and adherent cells were maintained with medium replenishment every 3 d. MSC were used from passage 2 to passage 5 in experiments.

### Animal model

Female C57BL/6 nude mice (6–7 weeks old) were purchased from the Department of Experimental Animals, Peking University Health Science Center. Animal experiments were performed according to the guidelines of the Institutional Animal Care and Use Committee at Peking University (Permit Number: 2011–0039). Mice were housed in a specific pathogen-free colony in the animal facility, and 100 μl of 2 × 10^6^ MDA-MB-231-FLUC cells suspended in saline were injected into the mammary fat pad to establish the orthotopic breast cancer model. After the tumor cell injection, the mice were given ZA (100 μg/kg) in 100 μl sterile phosphate-buffered saline (PBS) via daily tail vein injections (ZA group, *n* = 6). Control group (*n* = 6) was injected with equal volume of sterile PBS. The effect of ZA on tumor growth was examined by bioluminescence imaging (BLI).

To detect the effect of MSC on tumor growth, MDA-MB-231-FLUC human breast carcinoma cells were injected either alone, or coupled with MSC into the mammary fat pads of nude mice. MSC were treated with ZA (20 ng/ml) and cultured for 24 h or untreated. Tumor cell growth was evaluated by BLI. Mice were divided into three groups (*n* = 6 per group): MSC group, in which mice received co-injection of 1 × 10^6^ MSC and 1 × 10^6^ MDA-MB-231-FLUC cells in 100 μl of PBS; the ZA-MSC group, in which mice received the same amount of ZA-treated MSC and breast cancer cells; and the control (PBS) group received only PBS and cancer cells. Mice were matched for age and gender in each experiment.

To further determine the effect of MCP-1 in MSC tumor-promoting effect, MSC were transfected with MCP-1 siRNA or control siRNA. Tumor cell growth was detected by BLI at 10 d after establishing the breast carcinoma model. Animals were divided into three groups (*n* = 6 per group): MSC group, in which mice received injection of 1 × 10^6^ control siRNA-treated MSC and 2 × 10^6^ MDA-MB-231-FLUC cells in 100 μl of PBS; the si-MCP-1/MSC group, in which mice received 1 × 10^6^ treated MSC and 1 × 10^6^ MDA-MB-231-FLUC cells in 100 μl of PBS, the MSC were first treated by MCP-1 siRNA; and the PBS control group received 1 × 10^6^ MDA-MB-231-FLUC cells in 100 μl of PBS. Mice were matched for age and gender in each experiment.

### BLI

BLI was performed using the Xenogen IVIS Lumina II system (Perkin Elmer, Waltham, MA, USA) as detailed previously [27]. 8 min after intraperitoneal injection of D-Luciferin (150 mg/kg), the animals were imaged, and the same procedure was repeated at the specified time. Regions of interest (ROI) imaging signals were quantified in units of mean photons per second per square centimeter per steradian (p/s/cm^2^/sr).

### Real-time PCR

MSC were seeded onto 12-well plates (Corning, USA) at a density of 2 × 10^5^ cells/well and incubated overnight. The medium was replaced with a fresh medium containing ZA (20 ng/ml) and cultured for 24 h, after which real-time PCR analysis was performed. All experiments were performed in triplicate. To determine the effect of MCP-1 in MSC, MSC were transfected with MCP-1 siRNA in serum-free medium using Lipofectamin PLUS (Invitrogen, Carlsbad, CA). The sequences for MCP-1 siRNA were: Sense: 5′-AAUUGAUUUAGCGUACACGdTdT-3′; Antisense: 5′-CGUGUACGCUAAAUCAAUUdTdT-3′.

Total RNA was extracted from cell pellets using Trizol Reagent (Invitrogen) and treated with RNase-free DNase I (Qiagen, USA). First-strand cDNA synthesis was performed using ABI High-Capacity cDNA RT kit (Applied Biosystems, USA). Then, the RT-PCR and Real-time PCR were followed. Primers used in PCR were listed in Table 1, including interleukin-6 (IL-6), monocyte chemoattractant protein (MCP-1), macrophage inflammatory protein-1β (MIP-1β), tumor necrosis factor-α (TNF-α), interleukin-19 (IL-19), matrix metalloproteinase-2 (MMP-2), arginase type 1 (Arg-1), flammatory zone 1 (FIZZ-1), interleukin-10 (IL-10), CD47, and signal regulatory protein alpha (SIRPα). The total amount of mRNA was normalized to endogenous GAPDH mRNA. The Real-time PCR was performed in triplicate with the Fast Start Universal SYBR Green Master (ROX; Roche, Mannheim, Germany) and the iCycler iQ52.0 Standard Edition Optical System (Bio-Rad, Hercules, CA, USA).

**Table 1:** Primers used in Real-time PCR

Primers	Forward sequence (5′-3′)	Reverse sequence (5′-3′)
IL-6	TCCCCATCTCTCATGCAGTGT	CTCTCTCCCTTCTGAGCAGCTG
MCP-1	GTTGGCTCAGCCAGATGCA	CCAGCCTACTCATTGGGATCA
MIP-1β	CCCTGGGTCACTGAGTACATGA	CAAGGACGCTTCTCAGTGAGAA
TNF-α	CAGCCGATGGGTTGTACCTT	GTGTGGGTGAGGAGCACGTA
IL-19	GGTCTGGTTGGATCCCAATG	CCCATCCTTGATCAGCTTCCT
MMP-2	GGGGTCCATTTTCTTCTTCA	CCAGCAAGTAGATGCTGCCT
Arg-1	AGACAGCAGAGGAGGTGAAGAG	CGAAGCAAGCCAAGGTTAAAGC
FIZZ1	TGCTGGGATGACTGCTACTG	AGCTGGGTTCTCCACCTCTT
IL-10	TGCTAACCGACTCCTTAATGCA	TCATGGCCTTGAGACACCTTG
CD47	CCAAACTTTCCCCAGAACAG	AGGAGGAGAAAGGAGGTTGC
SIRPα	TGCAGTTGAGAATGGTCGAA	TCCGCGTCCTGTTTCTGTA
GAPDH	ACCTGCCAAGTATGATGACATCA	CCCTCAGATGCCTGCTTCAC

### Immunohistochemistry

Tumors were washed thoroughly in PBS and embedded in the optimal cutting temperature medium (OCT) (Sakura Finetek, Torrance, CA, USA). Cryosections (5–6 μm) were cut and stained with antibodies according to the manufacturer's protocol. To identify MSC, cells were stained with rat anti-mouse CD29 (BD Biosciences Pharmingen, San Diego, CA) and CD45 (BD Biosciences Pharmingen, San Diego, CA). To quantify TAMs recruitment, the tumor sections were stained with rat anti-mouse CD206 (BD Biosciences Pharmingen, San Diego, CA), the percentage of CD206-positive cells was determined by counting the number of cells in six random fields (400 × magnification) in three histology sections. Counting was performed by two blinded independent investigators. Alexa Fluor 594-conjugated donkey anti-rat secondary antibody (BD Biosciences Pharmingen, San Diego, CA) was applied appropriately. DAPI was used for nuclear counterstaining. To examine vascular density in tumor, rat anti-mouse CD31 antibody (BD Biosciences Pharmingen, San Diego, CA) was used. Capillary vessels were counted by an unbiased investigator in ten randomly selected high-power fields (HPF) by using a fluorescence microscope at 400 × magnification.

### Flow cytometry

For bone marrow-derived MSC surface marker analysis, MSC were suspended in staining buffer (PBS, 2% FBS) at a concentration of 2 × 10^6^ cells/ml, and the MSC suspension (100 μl) was incubated with fluorescent labeled rat anti-mouse CD29, CD73, CD90, CD105, CD34 and CD45 antibodies (BD Biosciences Pharmingen, San Diego, CA) for 30 min on ice. Cells were washed twice with staining buffer, and fluorescence intensity was measured by flow cytometry (FACSCaliber, BD Immunocytometry).

To measure macrophages infiltration in tumor sites, 10 days after transplantation of MSC and MDA-MB-231-FLUC cells, tumor tissue was minced and added to 10 ml of a 4 mg/ml solution of dispase (sigma-Aldrich) in DMEM (Invitrogen). The minced tissue and media were transferred to a 50-ml Erlenmeyer flask and incubated for 1 h at 37°C. Following the incubation, the tissue was filtered through 40 μm nylon cell strainer (BD Pharmingen) and washed twice in DMEM for FACS analysis (FACScan flow cytometer, Becton Dickinson). The tumor cells were stained with Alexa Fluor 647-conjugated rabbit anti-mouse CD206 (Abcam, Cambridge, MA) and FITC-labeled anti-mouse F4/80 (Abcam, Cambridge, MA).

### Statistical analysis

All data were expressed as the mean ± SEM. One-way analysis of variance (ANOVA) was used to determine intergroup differences. Least significant difference (equal variances) and Dunnett's T3 (non-equal variances) *post hoc* tests were used for testing the differences between groups. All tests were two-tailed, and differences were considered statistically significant at *P < 0.05.
